# Retraction: Episodic Memory: A Comparative Approach

**DOI:** 10.3389/fnbeh.2013.00184

**Published:** 2013-12-03

**Authors:** Frontiers in Psychiatry Editorial Office

The corresponding author (Gema Martin-Ordas) and the journal wish to retract the 11 June 2013 article cited above.

Based on information discovered after publication and reported to the journal in October 2013, this article was found to contain substantial sections that were taken verbatim without quotation and citation. The corresponding author, who was entirely responsible for the unintentional inclusion of this material, agrees with retraction of the article in its entirety and apologizes to the reviewers, editors, and readers of Frontiers in Behavioral Neuroscience for any adverse consequences that may have resulted from the article’s publication.

